# Comparative Study of the Fit Accuracy of Full-Arch Bar Frameworks Fabricated with Different Presintered Cobalt-Chromium Alloys

**DOI:** 10.1155/2018/1962514

**Published:** 2018-08-05

**Authors:** Hang-Nga Mai, Tae-Yub Kwon, Min-Ho Hong, Du-Hyeong Lee

**Affiliations:** ^1^Department of Prosthodontics, School of Dentistry, Kyungpook National University, 2177 Dalgubeoldae-ro, Jung-Gu, Daegu 41940, Republic of Korea; ^2^Department of Dental Biomaterials, School of Dentistry and Institute for Biomaterials Research & Development, Kyungpook National University, 2177 Dalgubeoldae-ro, Jung-Gu, Daegu 41940, Republic of Korea

## Abstract

**Purpose:**

This study was to measure the geometric discrepancies that occur during the sintering contraction of presintered Co-Cr alloys in a full-arch bar framework and to compare the variations between alloys from different manufacturers.

**Materials and Methods:**

Eighteen implant-supported full-arch bar frameworks were fabricated through a soft-machining process using presintered Co-Cr alloy blocks: Ceramill Sintron (CS), Soft Metal (SM), and Sintermetall (SML) (n=6 for each group). The sintered frameworks were digitized using a structured light scanner, and the scan images were superimposed on the reference design. The geometric discrepancies of the sintered frameworks were three-dimensionally analyzed for horizontal, angular, and internal discrepancies. Kruskal-Wallis and Mann–Whitney U tests were used to compare the discrepancies among the groups (*α*=.05).

**Results:**

Significant differences were found in the geometric discrepancy measurements among the groups. The CS group showed larger horizontal and angular discrepancies, followed by the SM and SML groups* (P*<.001). The root mean square (RMS) values for internal discrepancy were not statistically different among the groups (*P*=.778).

**Conclusion:**

The geometric discrepancies of full-arch bar frameworks fabricated using the soft-machining process were affected by accuracies in sintering contraction of presintered alloys.

## 1. Introduction 

The implant-supported bar overdenture is an effective prosthetic treatment for edentulous jaws, particularly when the implants are misaligned [1, 2]. Accuracy of a bar framework is essential for longevity of the treatment [2, 3]. Framework deformation during the fabrication procedure leads to inaccuracies and misfit of the frameworks [4]. The conventional fabrication method for dental prostheses is based on the lost wax technique and casting, and suitable casting alloys should be selected to meet clinical needs [5]. Currently, the cobalt-chromium (Co-Cr) alloy is commonly used for dental prosthesis frameworks [6]. The alloy has a reasonable cost and biocompatibility, as well as exhibiting good mechanical properties [5, 7]. The high modulus of elasticity enhances the stress distribution of the framework and decreases the thickness of the framework, thereby leaving more space for artificial teeth and denture base resin [5, 8].

The casting technique involves melting of the metal ingots and subsequent cooling for taking the shape of the framework [6, 8]. The liquefied metal inevitably shrinks during the cooling period [9]. The Co-Cr alloys have high melting ranges of the casting alloys [8]. As the solidus temperature increases, more contraction occurs [6]. This inherent feature of the casting technique makes it difficult to accurately fabricate full-arch frameworks using Co-Cr alloys in conventional processes. To offset the possible error of contraction process, the use of suitable investment materials and techniques has been recommended [9]. Nonetheless, when the framework is of a large size, the risk of framework misfit is considerably great due to unpredictable contraction of the alloy [6]. To avoid compromising the fit accuracy of the framework, the framework can be segmented and reconnected [2, 10]. However, the soldering adjustment decreases the homogeneity of the material and causes new errors in adaptation [11].

Computer-aided design and computer-aided manufacturing (CAD/CAM) are an alternative way to fabricate metal frameworks [12]. CAD/CAM involve fewer manual steps and enable a streamlined manufacturing process in combination with high predictability of the resultant products [13, 14]. The digital workflow is more efficient than conventional pathways in terms of time and cost benefits [15]. Either fully sintered or presintered metal alloys can be used for milling processes [16]. Fully sintered alloys are milled to the actual size of the frameworks using a hard-machining process [17, 18]. As there is no contraction after milling, hard machining has demonstrated more precision and predictability than the conventional casting method [14, 19]. However, the high degree of hardness of such metal alloys makes them more difficult to mill, which can, therefore, shorten the lifespans of tools and increase the maintenance costs of manufacturing devices [18]. Presintered metal alloys are milled to larger size than final one using a soft-machining process, followed by sintering of the milled prosthesis [19]. Because the hardness is lower in the presintered alloys than in the fully sintered alloys, soft machining is more time- and cost-effective than hard machining for producing prostheses [18, 20]. In addition, the risk of material contamination in the soft machining is low because the block is manufactured using a dry milling process [21].

Sintering of a presintered alloy is essential for achieving the full density and maximum strength of the material [22]. The alloy powder is finely distributed in a binder material [20]. During the sintering process, the binder material is burned off, and alloy powder particles are sintered without creating a fused phase [23]. This condensation process results in a decrease in volume—the sintering contraction of the milled products—of approximately 11% [20, 24, 25]. There have been studies evaluating fit accuracy of single crown and multiple-unit fixed dental prostheses (FDPs) fabricated using presintered metal alloys and soft machining [18, 19, 24, 25]. However, few articles have investigated the dimensional accuracy in full-arch bar frameworks fabricated by the presintered alloys. The purpose of this article was to measure the geometric discrepancies between the designed and sintered bar frameworks and to compare variations between alloy blocks from different manufacturers. The null hypothesis was that there was no difference in sintering contraction of presintered alloy blocks and, thus, selection of alloys would not affect the accuracy of the full-arch bar framework.

## 2. Materials and Methods

**Figure 1:**
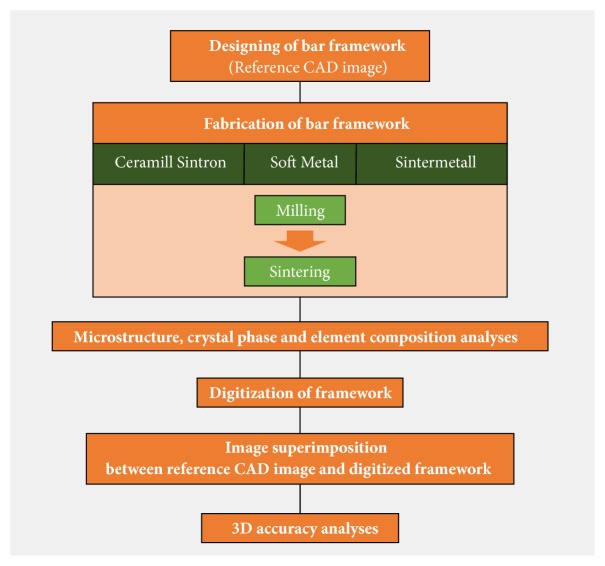
Workflow of this study.

**Figure 2:**
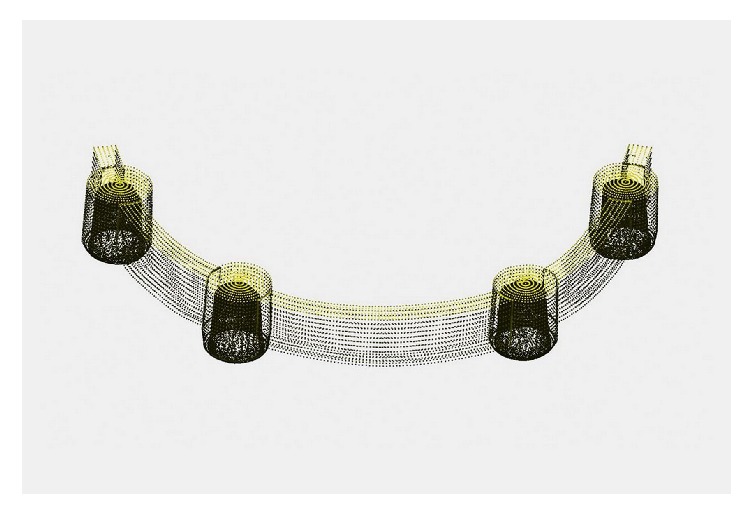
Computer-aided design image of bar framework.

The overall workflow of this study is described in Figure 1. A full-arch bar framework for an implant-supported overdenture was designed on an edentulous stone model with four implants using dental software (Ceramill Mind; Amann Girrbach AG, Koblach, Austria). The design was saved in standard tessellation language (STL) format as the reference image (Figure 2) and was then delivered to a 5-axis milling machine (Ceramill Motion 2; Amann Girrbach). Metal frameworks were dry-milled from three different presintered Co-Cr alloys: Ceramill Sintron (Amann Girrbach) (CS), Soft Metal (LHK, Chilgok, Korea) (SM), and Sintermetall (Zirkonzahn, South Tyrol, Italy) (SML). The milled frameworks were subsequently sintered to full density in the corresponding sintering furnaces. Information on the alloys and furnaces used is presented in Table 1. A total of 18-bar frameworks were fabricated (n=6 for each of the three groups), and all procedures were conducted following the manufacturers' instructions.

The microstructures of the fabricated specimens of each group were observed using optical microscopy (OM) analysis (MM-40/2U; Nikon, Tokyo, Japan). The crystal structures were evaluated using X-ray diffractometry (XRD) (MAXima-X XRD-7000; Shimadzu, Kyoto, Japan) with an accelerating voltage of 30 kV, a 2*θ* angle scan range of 30° to 100°, a beam current of 30 mA, a sampling pitch of 0.02°, a scanning speed of 2°/min, and a preset time of 0.6 s. For microstructural characterizations and element composition analyses, the specimens were examined by scanning electronic microscopy (SEM) (JSM-6700F; Jeol, Tokyo, Japan) with energy-dispersive X-ray spectroscopy (EDS) under an accelerating voltage of 15 kV.

**Figure 3:**
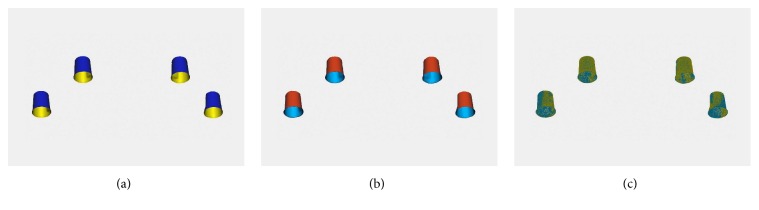
Internal surface of attachment component of framework. (a) Image segmented from reference design. (b) Image segmented from sintered framework. (c) Superimposed image of (a) and (b).

Geometric discrepancies in the metal frameworks were evaluated using three-dimensional (3D) analysis between the reference design and the sintered framework. No posttreatment was done on the surface of sintered frameworks. In the 3D analyses, the metal frameworks were digitized with a structured light scanner (Breuckmann smartScan; AICON 3D Systems GmbH, Braunschweig, Germany), and the internal surface image of the attachment component of the framework was segmented from the whole scan data of the bar framework. The same internal surface image was obtained from the reference design. Both images were superimposed using a best-fit registration algorithm function of the dental software package (Geomagic Design X; 3D Systems Inc., Morrisville, NC, USA) (Figure 3).

**Figure 4:**
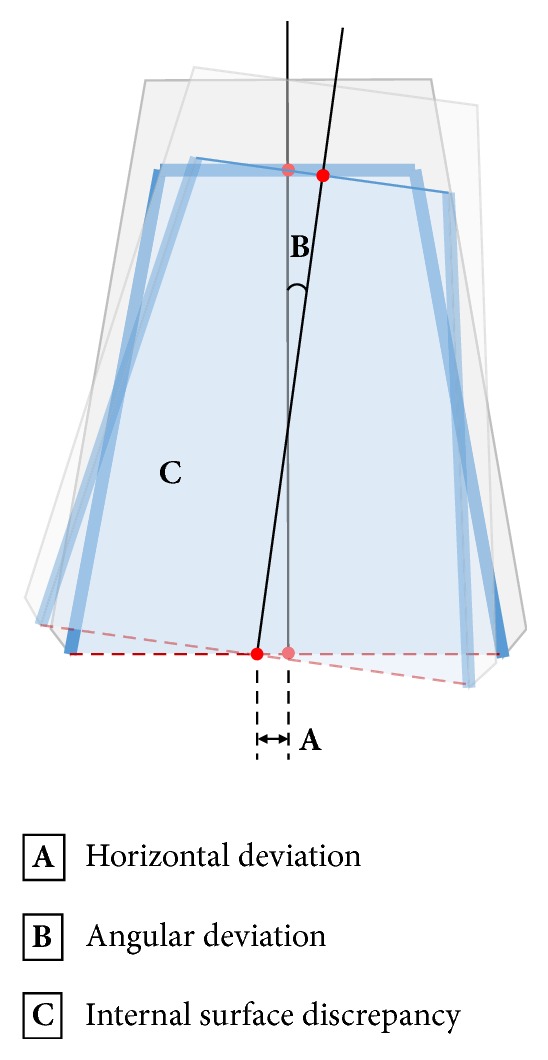
Measurement variables. A, horizontal discrepancy. B, angular discrepancy. C, internal surface discrepancy.

The outcome parameters for evaluating the geometric discrepancies of sintered frameworks were horizontal, angular, and internal surface discrepancies (Figure 4). The horizontal discrepancy was evaluated by measuring the distance between center points of each attachment component in horizontal plane view. The center point was defined as the intersection of the central longitudinal axis as it passed through the center of the base at a right angle to its plane. To determine angular discrepancy, the angles between the two centerlines of the attachment components from the reference design and the sintered framework were measured. The internal discrepancy between the reference image and the scan image was illustrated in a color-coded map, and the geometric discrepancies were computed for every data point. The root mean square (RMS) was calculated with the following formula [26]:

(1) $$\begin{array}{c} RMS=\sqrt{\frac{\sum_{i=1}^{n}{\left(x_{1,i}-x_{2,i}\right)}^{2}}{n}} \end{array}$$

where *x*_1,*i*_ is the measuring point *i* on reference image, *x*_2,*i*_ is the measuring point *i* on the scan image, and *n* is the total number of measuring points. The significance of discrepancies was illustrated in color codes as follows: green indicated a perfectly matched surface (error ± 30 *µ*m); yellow to orange shades indicated the test model was larger than the reference (error between + 30 *µ*m and +150 *µ*m); and light blue to dark blue shades meant the test model surface was smaller than the reference (error between -30 *µ*m and -150 *µ*m).

The measured values were compared among the groups. The mean and standard deviation (SD) values in micrometers were calculated for each group. The Kruskal-Wallis test was used to detect quantitative differences among the groups. The Mann–Whitney U test with Bonferroni correction was used to compare statistical differences between the groups (*α*=.05).

## 3. Results

**Figure 5:**
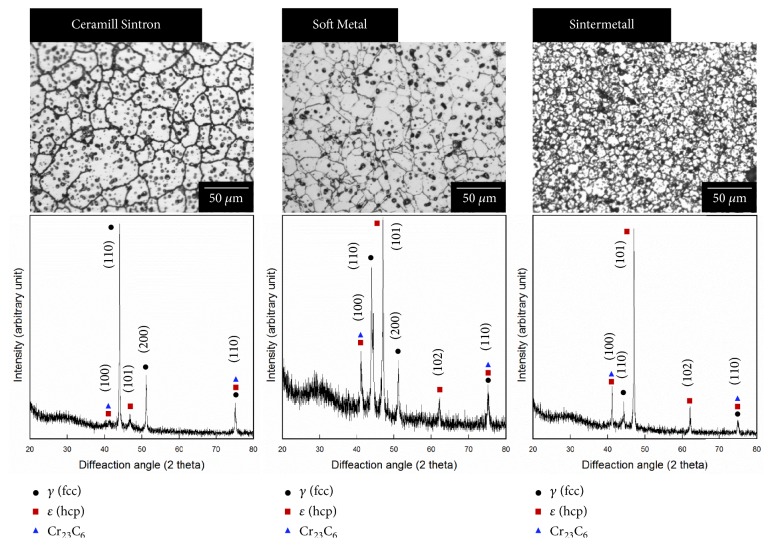
Optical microscopy images and corresponding XRD patterns of the Ceramill Sintron, Soft Metal, and Sintermetall specimens.

**Figure 6:**
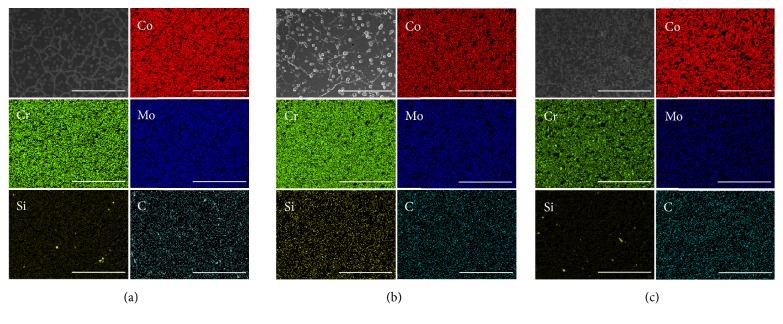
SEM and corresponding EDS mapping image for each group (500×, scale bar = 100*μ*m). (a) Ceramill Sintron. (b) Soft Metal. (c) Sintermetall.

Figure 5 shows OM images and XRD patterns of the presintered alloys from different manufacturers. Microstructures with round-shaped pores were observed in all samples of OM images. In addition, CS and SM groups showed similar grin sizes. However, SML group showed the finest microstructure compared to both groups. The XRD patterns of the 3 sintered alloys showed *γ* (face-centered cubic, fcc) and *ε* (hexagonal close-packed, hcp) matrix phases as well as Cr_23_C_6_ carbide. The Co-based *γ* (fcc) and *ε* (hcp) matrix phases were identified with ICDD cards no. 15-806 and no. 05-727, respectively. The peaks indexed as Cr_23_C_6_ metal carbides with a cubic structure were identified by ICDD card no. 35-783. The SEM and corresponding EDS mapping images of the all specimens tested were shown in Figure 6. All samples revealed homogeneous dispersion of individual element.

**Figure 7:**
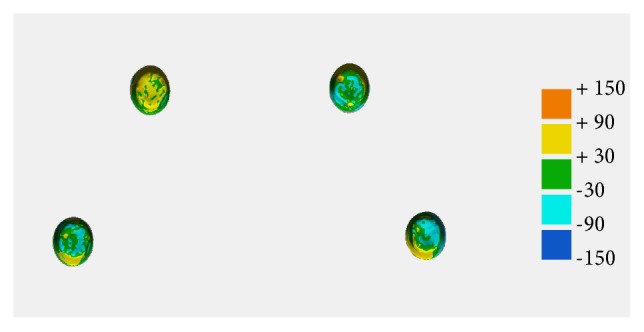
Color-coded map showing internal discrepancies in attachment component between reference design and sintered framework. Green indicates a perfectly matched surface (error ± 30 *µ*m). Yellow to orange shades indicate that the test model is larger than the reference design (error between +30 *µ*m and +150 *µ*m). Light blue to dark blue shades indicate that the test model surface is smaller than the reference design (error between -30 *µ*m and -150 *µ*m).

Table 2 presents the geometric discrepancies in the sintered frameworks of the three groups. The horizontal discrepancies in the framework were significantly different from each other (*P*<.001). The highest discrepancy was found for the CS group, followed by the SM and SML groups. The same tendency was found for the angular discrepancy. The CS group showed the highest discrepancy values (*P*<.001), and there was no significant difference between the SM and SML groups (*P*=.621). In terms of internal discrepancy, the mean RMS values were not statistically different among the groups (*P*=.778). The degree of discrepancy at specific points is illustrated in a color-coded map (Figure 7). In the SM and SML groups, most of the superimposed surface images were green shades, indicating that the reference design and the scan image corresponded well. The superimposed images of the CS group were represented by reddish or dark bluish ones, showing higher geometric differences with the reference design.

## 4. Discussion

This study evaluated the geometric discrepancies in full-arch bar frameworks fabricated by soft machining of presintered Co-Cr alloy blocks and compared the variations between different alloys. The result of this study showed that the horizontal and angular discrepancies of sintered frameworks differed according to the alloy used. Thus, the null hypothesis—that there is no difference in fabrication accuracy of the bar framework depending on the soft-machining system used—was rejected. The findings of this study correspond well with those found in an earlier accuracy study. Kim et al. [24] evaluated the marginal discrepancy of Co-Cr alloy copings fabricated by CAD/CAM techniques and stated that the marginal fit was material-specific in soft machining.

In an analysis of XRD pattern, alloys formed *γ* (face-centered cubic) and *ε* (hexagonal close-packed) matrix phases as well as Cr_23_C_6_ carbide. In particular, Ceramill Sintron showed a higher peak intensity of *γ* phase and Sintermetall showed a higher peak intensity of* ε *phase. These indicated that distinct forms were dispersed. Soft Metal was identified to create *γ* (face-centered cubic) and *ε* (hexagonal close-packed) matrix phases. In this way, different grain sizes and phase dispersion might be triggered by different final sintering temperatures depending on manufacturers.

The geometric discrepancies between the reference design and the sintered framework are due to contraction errors in the sintering process [18]. When frameworks are milled in presintered alloy blocks, they are formed larger than the final size [18, 22, 27]. The expansion ratio is determined by estimating the contraction ratio during the sintering process. Incorrect expansion cannot compensate for real contraction, leading to errors in the entire size [18]. Another error factor is the homogeneity of the presintered alloy block [22]. When the material composition is not homogeneous throughout the block, contraction may occur unevenly. This irregular contraction, depending on the area, causes distortion in large-scale structures [28]. Zhou et al. [29] also reported that a longer span length could lead to reduced adaptation of the framework. Previous articles on presintered alloys assessed the accuracy of prostheses in single or short-span FDPs and concluded that the use of presintered alloys was clinically acceptable [18, 24, 25]. Those findings imply that the general contraction ratio was precisely applied to the expansion ratio in milling. However, the accuracy is limited to a specific region of the block. On the other hand, the present study applied the presintered alloys to a full-arch framework design. Thus, it is possible that uniformity of contraction according to region was also evaluated in the geometric measurements. In other words, this study incorporated more possible sources of error in presintered alloy blocks by enlarging the framework size.

To pinpoint the deformation due to sintering contraction, irrelevant confounding factors were carefully controlled. First, each block from different manufacturers was trimmed using the same milling machine to eliminate effects of the milling process. Second, to minimize scanning errors, the sintered frameworks were digitized using a high-end structured light scanner with an accuracy level of 7 *µ*m [9, 30]. Third, the scan images of the sintered frameworks were directly compared with the reference designs using the best-fit superimposition algorithm in a certified 3D analysis software package [31]. In the literature, the accuracy of prostheses is generally evaluated by measuring the adaptation of a framework to the master cast [18, 24, 25]. Although this is a suitable method for verifying the fit accuracy of prostheses, it should be noted that some errors may occur when the prosthesis is placed and fixed on the master cast. Given the purpose of this present study, direct 3D analyses using the design and the scanned images may be optimal.

To the best of our knowledge, our results are the first reported data on 3D geometric discrepancies of full-arch frameworks stemming from sintering of presintered alloys. Various types of frameworks for multiunit FDPs and removable prostheses should be included in further studies to expand the application of presintered metal alloys. Moreover, large-scale clinical studies are vital for confirming the results of the in vitro studies and for including the number of clinical factors considered.

## 5. Conclusion

Horizontal and angular discrepancies of the full-arch framework that occurred during the sintering process were significantly different depending on the presintered Co-Cr alloys used. Special care should be taken in selecting presintered Co-Cr alloys for full-arch frameworks.

## Figures and Tables

**Table 1:** Co-Cr alloy systems used for fabricating the bar framework.

Co-Cr Alloy	Composition (weight %)^*∗*^	Sintering furnace	Manufacturer
Ceramill Sintron	Co 66, Cr 28, Mo 5, Si < 1, Fe < 1, Mn < 1	Ceramill Argotherm	Amann Girrbach, Koblach, Austria
Soft Metal	Co 63.4, Cr 29, Mo 5.8, Si 0.8, other elements < 1	Well-Burn (Denstar)	LHK, Daegu, Chilgok, Korea
Sintermetall	Co 65, Cr 27, Mo 5, C, N < 1	Sinterofen 300S	Zirkonzahn, South Tyrol, Italy

^*∗*^As provided by manufacturers.

C, carbon; Co, cobalt; Cr, chromium; Co-Cr, cobalt-chromium; Fe, iron; Mn, manganese; Mo, molybdenum; N, nitrogen; Si, silicon.

**Table 2:** Mean and standard deviation of geometric discrepancies of frameworks.

	Ceramill Sintron	Soft Metal	Sintermetall	*P*
Horizontal discrepancy (*µ*m)	94.8 (42.2)^a^	45.0 (19.2)^b^	28.5 (19.2)^c^	<.001
Angular discrepancy (degree)	1.4 (0.6)^a^	0.5 (0.3)^b^	0.4 (0.2)^b^	<.001
Internal discrepancy (*µ*m)	132.3 (70.8)	148.8 (50.7)	140.1 (42.2)	.778

Different superscript lowercase letters indicate significant differences within a row (*α*=.05).
